# Retraction: Differential temporal expression of S100β in developing rat brain

**DOI:** 10.3389/fncel.2023.1289247

**Published:** 2023-10-05

**Authors:** Frontiers Editorial Office

Following publication, concerns were raised regarding the integrity of the images in the published figures. The authors failed to provide a satisfactory explanation during the investigation, which was conducted in accordance with Frontiers' policies. As a result, the data and conclusions of the article have been deemed unreliable and the article has been retracted.

The retraction was approved by the Chief Editors of Frontiers in Cellular Neuroscience and the Chief Executive of Frontiers. The authors did not agree to the retraction.

